# Occurrence of *Fusarium* Mycotoxins and Their Modified Forms in Forage Maize Cultivars

**DOI:** 10.3390/toxins13020110

**Published:** 2021-02-02

**Authors:** Tim Birr, Tolke Jensen, Nils Preußke, Frank D. Sönnichsen, Marthe De Boevre, Sarah De Saeger, Mario Hasler, Joseph-Alexander Verreet, Holger Klink

**Affiliations:** 1 Department of Plant Diseases and Crop Protection, Institute of Phytopathology, Christian-Albrechts-University of Kiel, Hermann-Rodewald-Straße 9, 24118 Kiel, Germany; tolke-jensen@freenet.de (T.J.); javerreet@phytomed.uni-kiel.de (J.-A.V.); hklink@phytomed.uni-kiel.de (H.K.); 2 Otto Diels Institute for Organic Chemistry, Christian-Albrechts-University of Kiel, Otto-Hahn-Platz 4, 24118 Kiel, Germany; npreusske@oc.uni-kiel.de (N.P.); fsoennichsen@oc.uni-kiel.de (F.D.S.); 3 Centre of Excellence in Mycotoxicology and Public Health, Ghent University, Ottergemsesteenweg 460, 9000 Ghent, Belgium; Marthe.DeBoevre@UGent.be (M.D.B.); Sarah.DeSaeger@UGent.be (S.D.S.); 4 Lehrfach Variationsstatistik, Christian-Albrechts-University of Kiel, Hermann-Rodewald-Straße 9, 24118 Kiel, Germany; hasler@email.uni-kiel.de

**Keywords:** *Fusarium*, forage maize, modified mycotoxins, deoxynivalenol, deoxynivalenol-3-glucoside, 3-acetyl-deoxynivalenol, 15-acetyl-deoxynivalenol, zearalenone, α-zearalenol, cultivar

## Abstract

Forage maize is often infected by mycotoxin-producing *Fusarium* fungi during plant growth, which represent a serious health risk to exposed animals. Deoxynivalenol (DON) and zearalenone (ZEN) are among the most important *Fusarium* mycotoxins, but little is known about the occurrence of their modified forms in forage maize. To assess the mycotoxin contamination in Northern Germany, 120 natural contaminated forage maize samples of four cultivars from several locations were analysed by liquid chromatography-high resolution mass spectrometry (LC-HRMS) for DON and ZEN and their modified forms deoxynivalenol-3-glucoside (DON3G), the sum of 3- and 15-acetyl-deoxynivalenol (3+15-AcDON), α- and β-zearalenol (α-ZEL, β-ZEL). DON and ZEN occurred with high incidences (100 and 96%) and a wide range of concentrations, reaching levels up to 10,972 and 3910 µg/kg, respectively. Almost half of the samples (46%) exceeded the guidance value in complementary and complete feeding stuffs for ZEN (500 µg/kg), and 9% for DON (5000 µg/kg). The DON related mycotoxins DON3G and 3+15-AcDON were also present in almost all samples (100 and 97%) with amounts of up to 3038 and 2237 µg/kg and a wide range of concentrations. For the ZEN metabolites α- and β-ZEL lower incidences were detected (59 and 32%) with concentrations of up to 423 and 203 µg/kg, respectively. Forage maize samples were contaminated with at least three co-occurring mycotoxins, whereby 95% of all samples contained four or more mycotoxins with DON, DON3G, 3+15-AcDON, and ZEN co-occurring in 93%, together with α-ZEL in 57% of all samples. Positive correlations were established between concentrations of the co-occurring mycotoxins, especially between DON and its modified forms. Averaged over all samples, ratios of DON3G/DON and 3+15-AcDON/DON were similar, 20.2 and 20.5 mol%; cultivar-specific mean ratios ranged from 14.6 to 24.3 mol% and 15.8 to 24.0 mol%, respectively. In total, 40.7 mol% of the measured DON concentration was present in the modified forms DON3G and 3+15-AcDON. The α-ZEL/ZEN ratio was 6.2 mol%, ranging from 5.2 to 8.6 mol% between cultivars. These results demonstrate that modified mycotoxins contribute substantially to the overall mycotoxin contamination in forage maize. To avoid a considerable underestimation, it is necessary to analyse modified mycotoxins in future mycotoxin monitoring programs together with their parent forms.

## 1. Introduction

Maize (*Zea mays* L.) is one of the most important cereal crops worldwide and grown over a variety of climatic conditions. In the European Union (EU), maize was cultivated on about 15.3 million hectares in 2019, mainly utilizing as grain (8.9 million hectares) [1] and forage maize (aboveground whole plant; 6.4 million hectares) [2]. Grain maize serves as raw material for food, food components, feed, and biofuel, whereas forage maize is used as feedstuff or for biogas [3,4].

Maize as well as small-grain cereals like wheat, barley, and oats are susceptible to infections by fungi of the genus *Fusarium*. These fungi are known as plurivorous pathogens of various crops worldwide, which attack a range of plant parts and stages [5,6]. In European maize growing areas, *Fusarium* diseases of maize are caused by several co-occurring species, e.g., *F. graminearum*, *F. culmorum*, *F. avenaceum*, mainly associated with temperate and moist environmental conditions in North and Central Europe, and *F. verticillioides*, *F. temperatum*, *F. proliferatum*, commonly associated with warm and dry conditions in Southern European regions [6,7,8,9,10,11]. Infections of maize plants with phytopathogenic *Fusarium* species may occur throughout the whole vegetation period, whereby different pathways can be used to infect the maize plant, causing several rot diseases of ears and kernels (ear rot), rudimentary ears, roots, stems (stem rot), seeds, and seedlings [12].

Along with these infections, *Fusarium* mycotoxins are often produced and accumulated in affected tissues, which could pose a significant risk on human and animal health when entering the food and feed chain, because their occurrence in food and feed is often associated with mycotoxicosis. The toxigenic contamination of grain maize is caused by ear rot disease. As in forage maize production aboveground whole plants are harvested, forage maize includes all potentially *Fusarium*-infected and mycotoxin-contaminated organs of the maize plant such as rudimentary ears, stems, ears, and kernels [12]. Several *Fusarium* species are capable to produce a range of mycotoxins of diverse structures and actions, with deoxynivalenol (DON) and zearalenone (ZEN) being among the most important *Fusarium* mycotoxins in European maize production due to their frequent occurrence in partly high concentrations [13,14,15,16,17,18,19,20]. DON, also known as vomitoxin, is produced by *F. graminearum* and *F. culmorum* [6]. Exposure to DON induces gastrointestinal manifestations like vomiting and diarrhoea, feed refusal, anorexia, reduced growth and weight gain, and elicits immunological and neuroendocrine effects. Furthermore, DON is phytotoxic and, thus, considered as a virulence factor during fungal pathogenesis [21,22,23]. ZEN is mainly produced by *F. graminearum*, *F. culmorum*, *F. crookwellense*, and *F. equiseti* [6] and can be hepatotoxic and immunotoxic to a number of mammalian species. It is able to bind to oestrogen receptors due to its similarity to 17β-estradiol causing reproductive disorders, e.g., hyper-oestrogenism and infertility in livestock [22,24,25]. To avoid human and animal health risks, the European Union has defined maximum levels for DON and ZEN in foodstuffs [26] and guidance values for DON and ZEN in products intended for animal feeding [27].

In the last decade, attention to the occurrence of several, structurally closely related *Fusarium* mycotoxins in cereals and cereals-based food and feed, often called “modified mycotoxins”, has increased worldwide. The term “modified mycotoxin” refers to any mycotoxin whose chemical structure has been changed in the course of biological or chemical reaction, e.g., by microorganisms (fungi, bacteria), by the metabolism of plants or during food and feed processing [23,25]. The most abundant modified forms of DON and ZEN, which co-occur with their native forms, are deoxynivalenol-3-glucoside (DON3G), the acetylated derivatives 3-acetyl-deoxynivalenol (3-AcDON) and 15-acetyl-deoxynivalenol (15-AcDON), α-zearalenol (α-ZEL) and β-zearalenol (β-ZEL) [28,29]. DON3G, often described as “masked” DON, is produced as part of the plant defence system and, thus, represents a detoxification mechanism by the enzymatic conjugation of DON to a glucose moiety. This conjugation occurs in maize and other small-grain cereals like wheat and barley [30,31]. In contrast, 3- and 15-AcDON are considered as fungal derived metabolites occurring with DON especially in cereal commodities [28,31]. DON3G has a much lower intrinsic toxicity than its precursor [23,30,32], while 3- and 15-AcDON have at least equal or less intrinsic toxicity than DON (DON3G << 3-AcDON < DON ≈ 15-AcDON) [21,33,34]. The most abundant modified forms of ZEN are the hydroxylated forms α- and β-ZEL that can be formed by fungi, plants, and bacteria. Their occurrence has been described in several food and feed matrices [29,35]. Compared to ZEN, β-ZEL is less toxic, whereas α-ZEL demonstrates a significantly higher toxicity than the parent mycotoxin [24]. The occurrence of structurally related *Fusarium* mycotoxins is of increasing concern in food and feed safety, since it is assumed that modified mycotoxins can contribute substantially to the overall toxicity. An increase of toxic health effects by modified mycotoxins may be either direct or indirect by conversion (e.g., hydrolysis during digestion) into their parent forms [36,37].

Modified mycotoxins often remain undetected in commonly used commercial screening methods [23,31,38]. In the past few years, several studies have investigated the occurrence of modified mycotoxins such as DON3G, 3- and 15-AcDON, α- and β-ZEL in small-grain cereals like wheat [39,40,41,42,43,44,45,46,47], barley [41,43,48], oats [41,43] as well as grain maize [16,19,39,49,50]. In contrast to their frequent occurrence in partly high concentrations in the aforementioned small-grain cereals and grain maize, little is known about the occurrence of DON3G, 3- and 15-AcDON, α- and β-ZEL in freshly harvested forage maize. However, knowledge of the natural occurrence of these modified *Fusarium* mycotoxins together with their parent forms DON and ZEN in forage maize is essential due to the particular importance of ensiled forage maize (=maize silage) as an indispensable component in the diet of ruminants. Maize silage may constitute up to 75% of the daily forage dry matter intake of dairy cows and beef cattle [51] and is, therefore, a potential source of field-derived *Fusarium* mycotoxins DON and ZEN and their modified forms.

Our current study presents analytical data for DON and ZEN along with their modified forms in naturally contaminated forage maize samples collected at eight trial locations in Northern Germany from the 2017 harvest. One-hundred and twenty samples of four forage maize cultivars were analysed by liquid chromatography-high resolution mass spectrometry (LC-HRMS) in order to (i) obtain a more detailed picture of the co-occurrence of DON and ZEN and their modified forms DON3G, 3-AcDON, 15-AcDON, α-ZEL and β-ZEL in forage maize and (ii) determine the relative proportions of modified mycotoxins in relation to their measured parent forms.

## 2. Results

### 2.1. Occurrence of Deoxynivalenol and Zearalenone and Their Modified Forms in Forage Maize Samples

Forage maize samples of four cultivars, namely “SY Werena” (Cv I), “SY Kardona” (Cv II), “Colisee” (Cv III), and “Niklas” (Cv IV), were analysed for DON and ZEN and their modified forms DON3G, 3- and 15-AcDON (declared as sum of both = 3+15-AcDON), α- and β-ZEL. DON was the predominant *Fusarium* mycotoxin, being detected in all 120 samples of the four cultivars with a mean concentration of 2165 µg/kg and reaching high concentrations of up to 10,972 µg/kg (Table 1). ZEN was found in almost all samples (96% positive samples) with a mean concentration of 819 µg/kg and maximal contents of up to 3910 µg/kg. The guidance value for DON in complementary and complete feeding stuffs of 5000 µg/kg set in Commission Recommendation 2006/576/EC [27] was exceeded in 9% of all samples, whereas almost half of the samples (46%) contained ZEN concentrations higher than the guidance value of 500 µg ZEN/kg. The DON related forms DON3G and 3+15-AcDON were present in all (DON3G) or almost all samples (3+15-AcDON; 97% positive samples) with similar mean concentrations of 537 and 516 µg/kg and a maximum contamination of up to 3038 and 2237 µg/kg, respectively. Lower incidences and concentrations were detected for the ZEN metabolites α-ZEL and especially β-ZEL. In 59% of all samples, α-ZEL was present with a mean concentration of 44 µg/kg reaching maximum concentrations up to 423 µg/kg. In only 32% of all samples β-ZEL was detected, whereby almost all concentrations of these positive samples were below the detection capability (<CCβ). Therefore, the calculation of mean values was not applicable (Table 1).

In all four cultivars, DON reached significantly higher concentrations than any other mycotoxin, whereby no significant differences were observed between DON3G, 3+15-AcDON, and ZEN within a cultivar (Table 2). Concentrations of α-ZEL were significantly lower compared to the previously mentioned mycotoxins. The highest mean concentrations of DON were found in the cultivars “Cv I” and “Cv II” with 2486 and 2673 µg/kg, respectively, followed by the cultivar “Cv III” with 2054 µg/kg. Compared to the cultivars “Cv I” and “Cv II”, significantly lower DON concentrations were analysed in the cultivar “Cv IV” with a mean content of 1449 µg/kg. With 1055 µg/kg, the highest mean concentration of ZEN was detected in the cultivar “Cv II”. Lower but also high amounts were present in the remaining cultivars “Cv I”, “Cv III”, and “Cv IV” with mean concentrations of 785, 666, and 768 µg/kg, respectively. No significant differences in ZEN concentrations were determined between the four cultivars. The guidance value for DON of 5000 µg/kg was exceeded less frequently in all cultivars (3 to 13%) compared to the exceedance of the guidance value for ZEN of 500 µg/kg, ranging from 33 to 53% between cultivars (Table 2). Considerable amounts of the DON related form DON3G were detected in all four forage maize cultivars. Comparable mean concentrations were determined in the cultivars “Cv I” and “Cv III” with 507 and 526 µg/kg, respectively, whereas, as for DON, the highest concentrations were found in the cultivar “Cv II” with a mean concentration of 776 µg/kg. The lowest DON3G amounts were detected in the cultivar “Cv IV” with a mean contamination of 339 µg/kg, in which also the lowest DON concentrations were found. The sum of 3- and 15-AcDON (=3+15-AcDON) reached concentrations similar to DON3G with mean values of 472 µg/kg in the cultivar “Cv I”, 655 µg/kg in the cultivar “Cv II”, 559 µg/kg in the cultivar “Cv III”, and 378 µg/kg in the cultivar “Cv IV”. For DON3G and 3+15-AcDON significant cultivar-specific differences were determined between the cultivars “Cv II” and “Cv IV”. For DON, DON3G, 3+15-AcDON, and ZEN a wide range of concentrations between samples were determined in each cultivar. The ZEN metabolite α-ZEL occurred in all cultivars in more than half of the samples, but with much lower concentrations compared to the abovementioned mycotoxins. Mean concentrations varied from 31 to 64 µg/kg between the four cultivars. Concentrations of α-ZEL positive samples ranged from <CCβ to 423 µg/kg. A lower degree of contamination was found for β-ZEL in all cultivars. Incidences varied from 20 to 53% between cultivars, but almost all positive samples were below the detection capability (Table 2).

**Figure 1:**
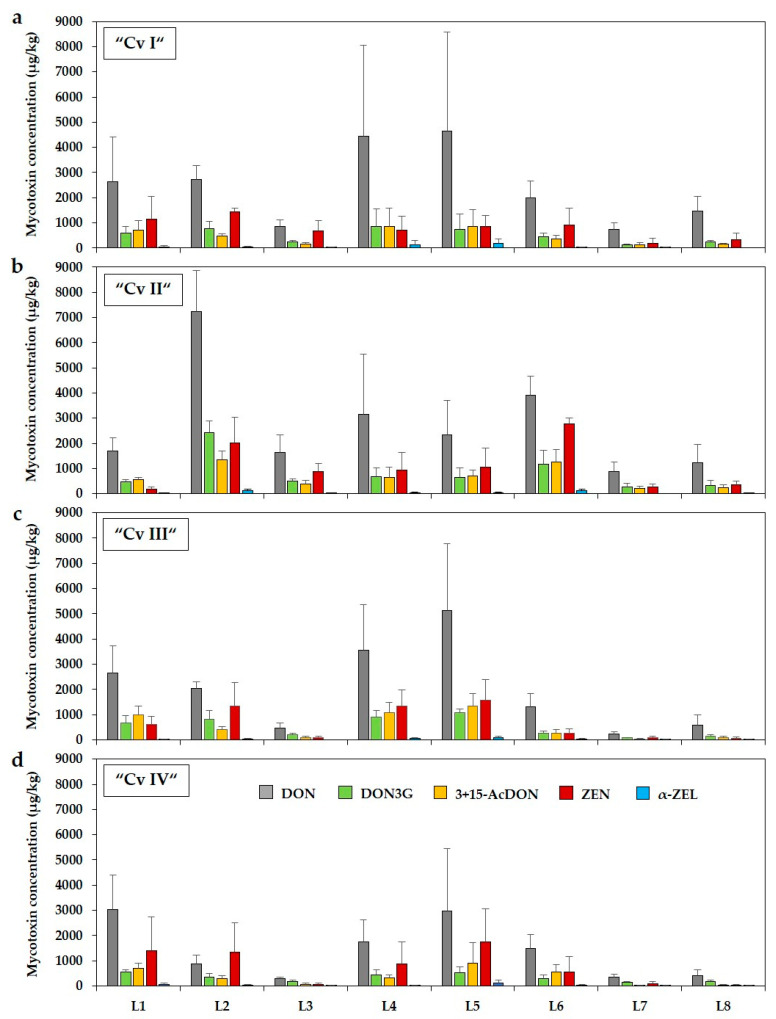
Mean concentrations (±SD) of DON, DON3G, 3+15-AcDON, ZEN, and α-ZEL (µg/kg) in forage maize samples of four different cultivars (Cv I–IV) (**a**–**d**) at eight trial locations (L1–L8). DON = deoxynivalenol; DON3G = deoxynivalenol-3-glucoside; 3+15-AcDON = sum of 3- and 15-acetyl-deoxynivalenol; ZEN = zearalenone; α-ZEL = α-zearalenol. *n* = 120.

As shown in Figure 1, concentrations of DON, DON3G, 3+15-AcDON, ZEN, and α-ZEL in forage maize samples of the four cultivars differed considerably between trial locations. In general, the highest mycotoxin concentrations were found at locations with pre-crop maize (L1, L2, L4, L5, L6; Table 4) compared to the other locations (L3, L7, L8) with pre-crops other than maize. Moreover, not only between locations a high variability of mycotoxin concentrations was found. A strong variability in *Fusarium* mycotoxin contamination was also observed between the individual samples (replications) within a location of each cultivar, which was reflected by high standard deviations.

### 2.2. Co-Occurrence and Correlations between Fusarium Mycotoxins

**Figure 2:**
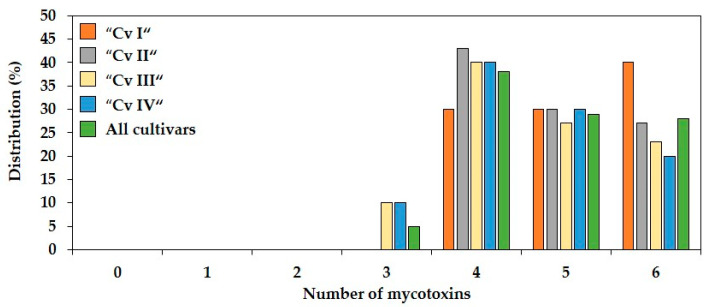
Distribution (%) of the number of *Fusarium* mycotoxins in forage maize samples of four different cultivars (Cv I–IV). *n* = 120.

Due to the high incidences of most of the six investigated *Fusarium* mycotoxins (DON, DON3G, 3+15-AcDON, ZEN, α-ZEL, β-ZEL) a minimum of three co-occurring mycotoxins was found in forage maize samples of the four cultivars. Only 5% of all samples contained three mycotoxins, whereas the remaining 95% of the 120 samples were contaminated with four or more mycotoxins (Figure 2). Four co-occurring mycotoxins were present in 38%, five mycotoxins in 29% and six mycotoxins in 28% of all samples. The frequencies of the co-occurring mycotoxins in the different forage maize cultivars were similar.

**Figure 3:**
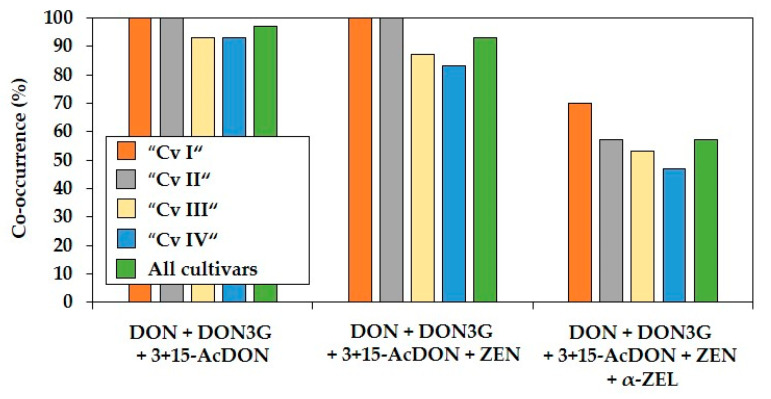
Percentages of most co-occurring *Fusarium* mycotoxin combinations containing three, four, and five mycotoxins in forage maize samples of four different cultivars (Cv I–IV). DON = deoxynivalenol; DON3G = deoxynivalenol-3-glucoside; 3+15-AcDON = sum of 3- and 15-acetyl-deoxynivalenol; ZEN = zearalenone; α-ZEL = α-zearalenol. *n* = 120.

Regarding different combinations of co-occurring mycotoxins, DON and its related forms DON3G and 3+15-AcDON co-occurred as the most frequent triple combination of mycotoxins in 97% of all forage maize samples (Figure 3), demonstrating a strong co-occurrence of DON and its modified forms. The most frequent mycotoxin combination with a minimum of four co-occurring mycotoxins was the combination of DON + DON3G + 3+15-AcDON + ZEN, being present in 93% of all samples ranging from 83 to 100% between the four cultivars. Together with α-ZEL, these four *Fusarium* mycotoxins represented the most frequent combination of five co-occurring mycotoxins in all samples with a percentage of 57%. The maximum combination of six mycotoxins containing DON, ZEN, DON3G, 3+15-AcDON, α- and β-ZEL was detected in 28% of all samples (Figure 2). 

Additionally, a correlation analysis between individual mycotoxin concentrations was performed (Table 3). All pairwise correlations between DON, DON3G, 3+15-AcDON, ZEN, and α-ZEL (β-ZEL not considered) were positive, although concentrations of these mycotoxins fluctuated considerably between locations and replications as shown in Figure 1. Highly positive correlations were found in all four cultivars between concentrations of DON and DON3G (all cultivars: *r* = 0.886), DON and 3+15-AcDON (all cultivars: *r* = 0.915), DON3G and 3+15-AcDON (all cultivars: *r* = 0.841), demonstrating a strong relationship of concentrations between DON and its modified forms in forage maize samples (Table 3). Compared to this, weaker positive correlations were observed between ZEN and DON (all cultivars: *r* = 0.714) and its related forms DON3G and 3+15-AcDON, between ZEN and its metabolite α-ZEL (all cultivars: *r* = 0.619), and between α-ZEL and DON, DON3G and 3+15-AcDON.

### 2.3. Ratios between DON3G and DON, 3+15-AcDON and DON, α-ZEL and ZEN

**Figure 4:**
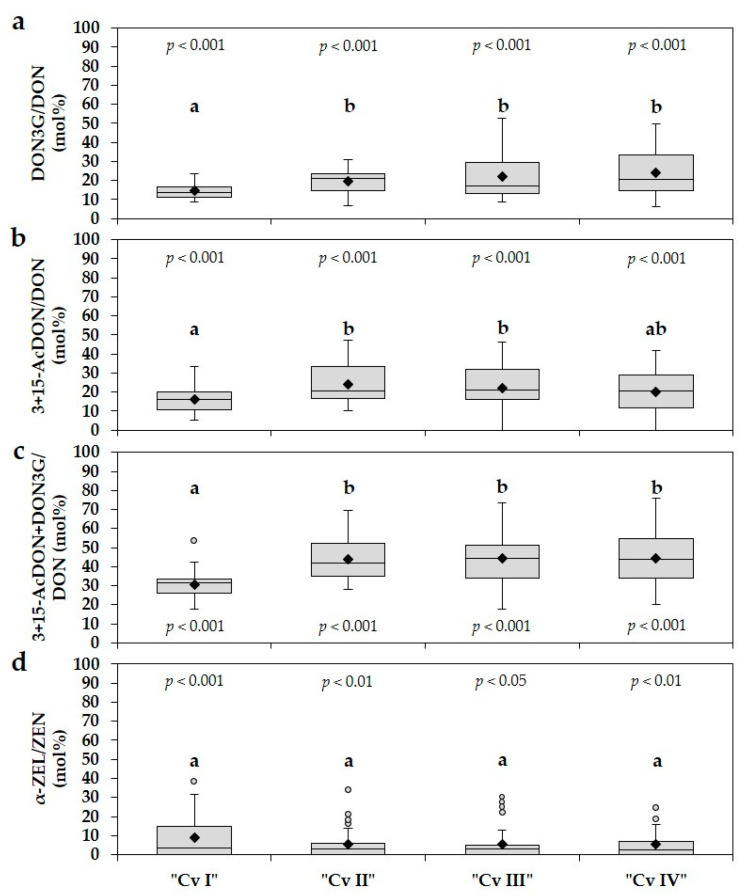
Boxplots and means (black rhombus) of ratios between (**a**) DON3G and DON, (**b**) 3+15-AcDON and DON, (**c**) 3+15-AcDON + DON3G and DON, (**d**) α-ZEL and ZEN (mol%) in forage maize samples of four different cultivars (Cv I–IV). Five statistics are represented in each boxplot from bottom to top: the smallest observation, lower quartile, median, upper quartile, and largest observation, respectively. Different letters describe significant differences between cultivars for each ratio (*p* ≤ 0.05). The *p*-values describe significant or insignificant differences of mean ratios from zero. DON = deoxynivalenol; DON3G = deoxynivalenol-3-glucoside; 3+15-AcDON = sum of 3- and 15-acetyl-deoxynivalenol; ZEN = zearalenone; α-ZEL = α-zearalenol. *n* = 120.

The relative proportions of DON3G and 3+15-AcDON to DON, and α-ZEL to ZEN in the four forage maize cultivars are indicated in Figure 4. As the molecular masses of 3- and 15-AcDON (338.35 g/mol) and especially DON3G (458.46 g/mol) are higher than the molecular mass of DON (296.31 g/mol), data were converted from mass to molar concentrations and the ratios of the abovementioned proportions are given in mol%. For the calculation of α-ZEL/ZEN ratios, the molecular masses of both mycotoxins were also considered, although both masses are very similar (ZEN: 318.36 g/mol; α-ZEL: 320.39 g/mol). As a small part of forage maize samples did not contain any ZEN, only ZEN positive samples were considered for the calculation of α-ZEL/ZEN ratios.

The concentrations of DON3G in individual forage maize samples ranged from 6.4 to 52.7 mol% relative to their DON concentrations (Figure 4a). Averaged over the four cultivars, 20.2 mol% of the measured DON concentration was found as DON3G. The lowest mean DON3G/DON ratio was observed in the cultivar “Cv I” with 14.6 mol%, whereas significantly higher ratios were determined in the remaining cultivars “Cv II”, “Cv III”, and “Cv IV” with 19.8, 22.2, and 24.3 mol%, respectively. The ratio 3+15-AcDON/DON varied from 0 to 47.3 mol% between samples (Figure 4b). Concentrations of 3+15-AcDON reached 20.5 mol% relative to their DON concentrations averaged over the four cultivars, with comparable mean ratios as shown before for the DON3G/DON ratio ranging from 15.8 to 24.0 mol% between cultivars. The summarized ratio 3+15-AcDON + DON3G/DON reached a mean level of 40.7 mol% averaged over all cultivars. Significantly higher mean ratios were determined for the cultivars “Cv II”, “Cv III”, and “Cv IV” with 43.9, 44.4, and 44.2 mol%, respectively, compared to the cultivar “Cv I” with 30.4 mol% (Figure 4c). The ratios between samples ranged from 17.6 to 76.0 mol%. All single means of the three ratios DON3G/DON, 3+15-AcDON/DON, and 3+15-AcDON + DON3G/DON of the four cultivars were significantly different from zero. Hence, with a probability of 95% the additional DON in form of DON3G, 3+15-AcDON and the sum of both was not caused by chance (Figure 4a–c). The abovementioned ratios demonstrate that in average almost half of the measured parent DON concentrations were present in the modified DON forms DON3G and 3+15-AcDON in forage maize samples of the cultivars “Cv II”, “Cv III”, and “Cv IV”; about one-third in the cultivar “Cv I”. In all four cultivars no clear relationship between measured DON concentrations and ratios of DON3G/DON and 3+15-AcDON/DON was found (Figure S1). Increasing DON concentrations did not result in increasing or decreasing ratios of DON3G/DON and 3+15-AcDON/DON. In contrast, lower ratios of α-ZEL/ZEN were determined compared to the abovementioned ratios (Figure 4d). Concentrations of α-ZEL ranged from 0 to 39.1 mol% relative to their ZEN concentrations with a mean ratio of 6.2 mol% averaged over the four cultivars. Similar mean ratios were observed between cultivars ranging from 5.2 to 8.6 mol%. Although a wide range of α-ZEL/ZEN ratios was observed, ratios of most individual samples of each cultivar were below the mean value or equal to zero (Figure 4d, Figure S2). Furthermore, most of the highest α-ZEL/ZEN ratios were determined in samples which contain lower ZEN concentrations (Figure S2).

### 2.4. Total Concentrations of DON and ZEN by Considering DON3G, 3+15-AcDON and α-ZEL

**Figure 5:**
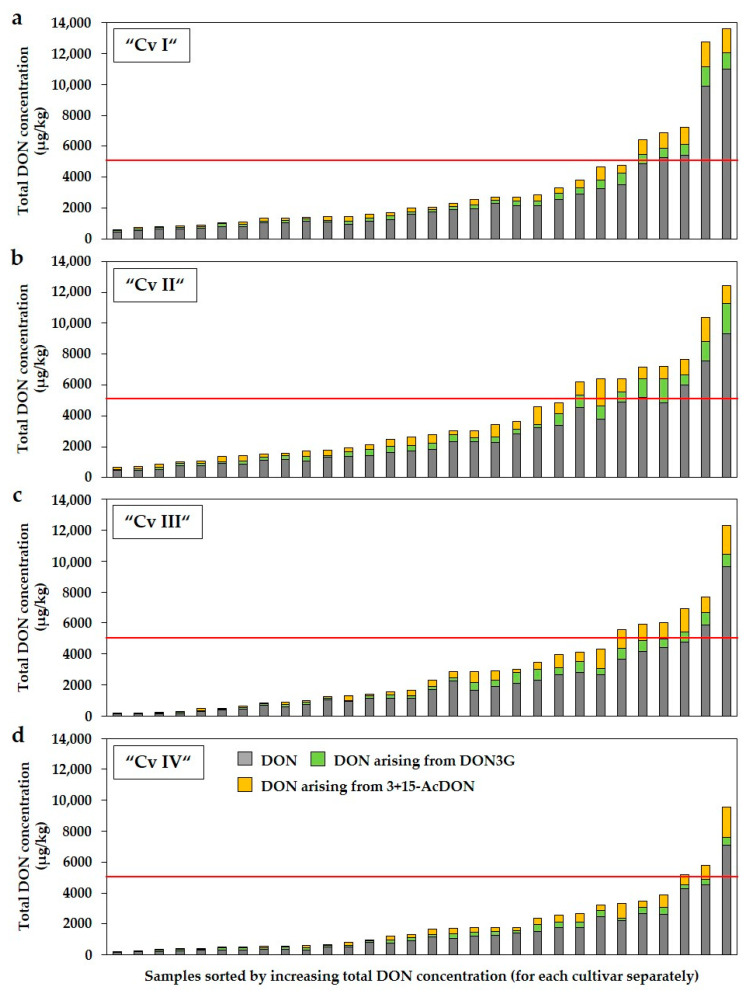
Total DON concentrations (µg/kg) in forage maize samples of four different cultivars (Cv I–IV) (**a**–**d**) by considering DON3G and 3+15-AcDON. The concentration of DON + DON3G + 3+15-AcDON of each sample was calculated by adding up all concentrations of the measured DON and DON arising from DON3G and 3+15-AcDON assuming a total conversion of DON3G and 3+15-AcDON into parent DON. For the determination of DON concentrations arising from DON3G and 3+15-AcDON concentrations of DON3G and 3+15-AcDON were normalised to the molecular mass of DON. Samples were sorted by increasing concentration of DON + DON3G + 3+15-AcDON for each cultivar separately. The guidance value for DON in complementary and complete feeding stuffs (5000 µg/kg) [27] is shown as red line. DON = deoxynivalenol; DON3G = deoxynivalenol-3-glucoside; 3+15-AcDON = sum of 3- and 15-acetyl-deoxynivalenol. *n* = 120.

Total concentrations of DON were calculated for all forage maize samples of the four cultivars by adding up all concentrations of DON and DON arising from DON3G and 3+15-AcDON assuming a conversion of DON3G and 3+15-AcDON into parent DON (Figure 5). It is important to note that concentrations of DON3G and 3+15-AcDON were normalised to the molecular mass of DON in order to calculate total DON concentrations. 

Relatively low DON concentrations found in the analysed samples of the four cultivars suggest a relative low risk from the feed safety point of view by additional DON arising from DON3G and 3+15-Ac-DON (Figure 5). In contrast, the risk is potentially increased by additional DON in samples with increasing DON concentrations, especially if the measured DON concentrations alone (grey part of the bars) reached higher values near the guidance value. For the analysed DON concentrations alone, the guidance value in complementary and complete feeding stuffs (5000 µg/kg) was exceeded in 9% (11 of 120) of all samples of the four cultivars, whereas the guidance value was exceeded in 18% (23 of 120) of all samples when the additional DON of DON3G and 3+15-AcDON were taken into account (grey, green, and yellow parts of the bars together). In the cultivar “Cv I”, the exceedance of the guidance value increased from 13% (4 of 30 samples) to 17% (5 of 30), in the cultivar “Cv II” from 13% (4 of 30) to 27% (8 of 30), in the cultivar “Cv III” from 7% (2 of 30) to 20% (6 of 30), and in the cultivar “Cv IV” from 3% (1 of 30) to 10% (3 of 30). Compared to DON and its modified forms, considering α-ZEL did not result in any further exceedance of the guidance value for ZEN in complementary and complete feeding stuffs of 500 µg/kg (Figure S3). α-ZEL was present in low concentrations in relation to its parent form. Nevertheless, the guidance value for ZEN was exceeded in almost half of all samples.

## 3. Discussion

Forage maize is an indispensable component in the diet of ruminants such as dairy cows and beef cattle. Freshly harvested whole-crop forage maize is commonly conserved by ensiling before being fed to ruminants, whereby ensiled forage maize (=maize silage) comprises up to 75% of the daily forage dry matter intake [51,52]. Forage maize may contain mycotoxins originating from pre-harvest contamination by fungi of the genus *Fusarium* that adversely affects the performance and health of livestock [53]. It is well known that the co-occurring mycotoxins DON and ZEN are one of the most important *Fusarium* mycotoxins in maize production due to their frequent occurrence in toxicologically relevant concentrations [54]. In contrast, little is known about the natural occurrence of modified forms of DON and ZEN in forage maize. Since it is assumed that these modified mycotoxins represent a serious health risk to exposed animals, knowledge of their natural occurrence in forage maize is of increasing concern in feed safety. In our present study, we investigated the occurrence of DON and ZEN along with their major modified forms DON3G, 3- and 15-AcDON (sum of both = 3+15-AcDON), α- and β-ZEL in 120 naturally contaminated forage maize samples of four cultivars collected at eight trial locations in Northern Germany from the 2017 harvest in order to obtain a more comprehensive picture of the natural co-contamination of forage maize with DON and ZEN and their modified forms.

DON and ZEN occurred with high incidences being present in all (DON) or almost all forage maize samples (ZEN; 96%) with mean concentrations of 2165 and 819 µg/kg, reaching high contents up to 10,972 and 3910 µg/kg, respectively. The frequent co-occurrence and the partly high contamination levels of DON and ZEN are consistent with previous studies in forage maize of Oldenburg and Höppner [55], and Oldenburg and Ellner [13] in Germany, Eckard et al. in Switzerland [56], Kosicki et al. in Poland [57], and Vandicke et al. in Belgium [10]. Based on the results of our and other studies and the fact that ensiled forage maize is a major component of the dairy cattle diet [51], it can be expected that forage maize is a major dietary source of DON and ZEN. Notably, the guidance value for ZEN of 500 µg/kg in complementary and complete feeding stuffs was exceeded in almost half of the samples tested here (46%), whereas an exceedance of the guidance value for DON of 5000 µg/kg was determined in 9% of all samples.

Several agronomic practices such as cultivar susceptibility, crop rotation, and soil cultivation are known to influence the occurrence of *Fusarium* species and, therefore, the level of mycotoxin contamination [58]. The maize cultivar influenced DON accumulation significantly in our study, which is in line with results of Oldenburg and Höppner [55] in forage maize, Lauren et al. [59], De Boevre et al. [19], and Mitsuhashi et al. [50] in grain maize. In contrast, no significant differences were observed in ZEN accumulation between the four investigated cultivars. This can be explained by the fact that breeding programs mainly focus on developing cultivars with a low DON accumulation [19]. Unfortunately, susceptible classifications of maize cultivars towards *Fusarium* infections are so far available for only a limited number of forage maize cultivars [60]. From our four investigated cultivars only the cultivar “Cv III” (“Colisee”) is classified (lowly susceptible). Furthermore, the different susceptibility levels are only related towards stem rot disease, not towards ear rot disease [60], which is of major importance in affecting the toxicogenic quality of grain-based products as well as forage maize used for human or animal nutrition [12]. Regarding the effect of crop rotation, locations with pre-crop maize and high proportions of maize in crop rotation showed a strong tendency to higher contamination with DON and ZEN in all four cultivars compared to locations with pre-crops other than maize. *Fusarium* pathogens saprophytically survive as mycelia in infected crop residues after harvest. These residues serve as a source of inoculum on which *Fusarium* species generally produce asexual conidia, but some also produce sexual ascospores (e.g., *F. graminearum*). Both are dispersed within the following crop by rain-splashing or wind, causing infections of several plant parts (e.g., ears, rudimentary ears, stems). *Fusarium* epidemics and, therefore, higher mycotoxin concentrations are favoured when maize cultivation follows host crops including small-grain cereals and especially maize. Additionally, the risk of *Fusarium* infections increases by cultivating maize in reduced-cultivation tillage systems that preserve high amounts of residues from the previous crop on the soil surface [58,61,62,63].

In addition to the frequent co-occurrence of DON and ZEN, our results also demonstrate that the modified mycotoxins DON3G, 3- and 15-AcDON, and α- and β-ZEL can contribute substantially to the overall mycotoxin contamination in forage maize along with their parent forms, which is in line with other studies in grain maize and small-grain cereals regarding a natural *Fusarium* mycotoxin contamination [16,19,39,41,45,49,64]. In contrast, there are only a few studies on the occurrence of modified forms of DON and ZEN in forage maize, mainly considering 3- and 15-AcDON [10,13,56]. In our study, the plant metabolite DON3G was present in all samples with partly high concentrations of up to 3038 µg/kg. Likewise, almost all forage maize samples contained the mono-acetylated DON derivatives 3- and 15-AcDON (sum of both; 97% positive samples) with significant amounts, reaching maximum concentrations of 2237 µg/kg. These results are in line with findings of Oldenburg and Ellner [13], and Vandicke et al. [10], who also reported the presence of 3- and 15-AcDON in forage maize samples originating from Germany and Belgium. The hydroxylated ZEN metabolite α-ZEL was determined with a considerable incidence of 59% positive samples in our study, but with lower concentrations compared to the abovementioned mycotoxins, although a higher contamination was observed in some samples with maximum levels up to 423 µg/kg. Much lower incidences of α-ZEL in forage maize samples were found by Oldenburg and Ellner [13] in a *Fusarium* mycotoxin monitoring in Germany in 2000 and 2002. Compared to α-ZEL, β-ZEL was detected less frequently with 32% positive samples and low concentrations; most of them below the detection capability. Due to the high incidences of most investigated mycotoxins, our findings indicate that a multi-mycotoxin contamination must be expected in forage maize consisting of parent mycotoxins and its modified forms. A strong co-occurrence was especially observed for DON and its related forms DON3G and 3+15-AcDON, co-occurring in 97% of all forage maize samples. Furthermore, highly positive correlations were established between concentrations of DON and its modified forms in all four cultivars. Therefore, our findings demonstrate that increasing concentrations of DON are coupled with increasing concentrations of DON3G and 3+15-AcDON, which was also reported in grain maize and small-grain cereals [19,47,50,65]. Moreover, DON and its modified forms also co-occurred with ZEN in almost all samples (93%). Correlation analysis showed positive relationships, especially between DON and ZEN [19,57]. This is due to the fact that DON and ZEN are produced by the same *Fusarium* species (*F. graminearum* and *F. culmorum*) [6]. In addition, more than half of all forage maize samples (57%) were co-contaminated with DON, its modified forms DON3G and 3+15-AcDON, ZEN and α-ZEL. The co-contamination with multiple mycotoxins and, therefore, the exposure to a larger number of toxic compounds may be more serious due to potential synergistic or additive effects on livestock health compared to the presence of a single mycotoxin [66,67].

Modified mycotoxins often remain undetected in commonly used commercial screening methods [23,31,38], which only consider parent mycotoxins. Consequently, their occurrence would potentially imply an underestimation of the overall mycotoxin contamination in forage maize.

In our study, concentrations of DON3G reached 20.2 mol% relative to their measured parent DON concentrations averaged over all 120 forage maize samples, i.e., that the DON contamination was underestimated by 20.2 mol% when DON3G would not have been detected. Comparable mean ratios of DON3G/DON were obtained by other researchers, but in other crops like wheat or maize utilisations such as grain maize. Ratios of 14 and 17 mol% were observed by Berthiller et al. [39] in grain maize and wheat grain. Bryla et al. [47] determined also similar mean ratios of DON3G/DON of 22 mol% in wheat grain, while Wei et al. [49] found comparable proportions in grain maize with 25 mol%. In a study of Nathanail et al. [41], also comparable ratios were observed with 19 mol% in oat grain and 14 mol% in wheat grain. However, our results demonstrate that in some cases DON3G/DON ratios can reach much higher values in individual samples of up to 52.7 mol% in forage maize, confirming the results of other studies in grain maize and wheat grain [39,47,49]. Furthermore, DON3G/DON ratios seem to be cultivar-specific as described by Amarasinghe et al. [42] and Lemmens et al. [44] in wheat grain after ear inoculation with *F. graminearum*, Tucker et al. [48] in barley grain after ear inoculation with *F. graminearum*, and Mitsuhashi et al. [50] in grain maize after silk inoculation with *F. graminearum*. Their investigations showed that cultivars with a higher level of *Fusarium* resistance (lower DON accumulation) were characterized by an elevated ratio of DON3G/DON due to a higher ability to convert DON into DON3G in phase II metabolism of plants compared to more susceptible cultivars (higher DON accumulation). Moreover, in our study the forage maize cultivars “Cv III” and “Cv IV” with a lower DON accumulation (2054 and 1449 µg/kg, respectively) were characterized by a significantly higher DON3G/DON ratio of 22.2 and 24.3 mol% compared to the more susceptible cultivar “Cv I” (14.6 mol%) with a higher DON contamination (2486 µg/kg). In contrast, no significant differences in DON3G/DON ratios were determined between the cultivars “Cv II”, “Cv III”, and “Cv IV”, although the highest DON accumulation was found in the cultivar “Cv II” (2673 µg/kg). Therefore, further investigations are necessary to analyse the DON3G/DON ratio of a broader spectrum of different *Fusarium* susceptible forage maize cultivars. Nevertheless, it is assumed that an increased cultivation of maize and small-grain cultivars with an increased *Fusarium* resistance, which is coupled with an increased ability to conjugate DON, may lead to a higher DON3G/DON ratio [39]. In consequence, a higher underestimation of the actual mycotoxin contamination must be expected when considering only parent DON in mycotoxin analyses in cultivars with higher DON3G/DON ratios. Furthermore, our results indicate that increasing DON concentrations did not result in a decreasing DON3G/DON ratio as reported by Ovando-Martínez et al. [40] in wheat, who observed a lower DON3G/DON ratio in samples with higher DON concentrations (>30,000 µg/kg).

A very similar ratio was determined between 3+15-AcDON and parent DON averaged over all forage maize samples. 3+15-AcDON contribute 20.5 mol% to the measured DON contamination when considered. Vandicke et al. [10] determined a mean ratio of 26% between 3+15-AcDON and DON in forage maize on a mas basis. This corresponds to a molar ratio of 23 mol%; similar to the ratio determined in our study. Comparable ratios of 3+15-AcDON/DON were observed also in grain maize by Schollenberger et al. [14], Goertz et al. [16], and De Boevre et al. [19]. Our correlation analysis showed a strong relationship between concentrations of 3+15-AcDON and DON in all four cultivars indicating a proportional production in forage maize. This is due to the fact that DON and its acetylated derivatives 3- and 15-AcDON are produced by fungi, especially *F. graminearum* and *F. culmorum* [6,28]. Both, 3- and 15-AcDON, arise from the precursor 3,15-diacetyl-deoxynivalenol and are thus biosynthetic precursors of DON [28].

Taken together, 40.7 mol% of the measured parent DON was present in form of DON3G and 3+15-AcDON averaged over all samples and cultivars, which remain undetected in most routine mycotoxin analytical methods resulting in a considerable underestimation of the total mycotoxin contamination. Moreover, their occurrence not only increases the overall mycotoxin accumulation in forage maize. Their occurrence also increases the complexity of feed safety problems since modified forms can contribute substantially, either directly or indirectly, to the overall toxicity (e.g., by conversion into their parent forms) [23,36,37]. The acetylated forms 3- and 15-AcDON are equivalently or less toxic than DON (3-AcDON < DON ≈ 15-AcDON) [21,33,34] and both are for the most part de-acetylated into DON during absorption and distribution by animals [33,68]. In contrast, toxicity studies all agree that the toxic potency of DON3G is much lower compared to its parent DON [32]. DON3G is formed in planta by enzymatic conjugation of DON with glucose, representing a detoxification mechanism of parent DON in plants [30,31]. However, it was shown by Berthiller et al. [36] that several intestinal lactic acid bacteria (LAB) have the capability to hydrolyse the DON3G conjugate, releasing its toxic precursor mycotoxin DON during mammalian digestion. In case of 3-and 15-AcDON, it is known that certain anaerobic bacterial strains are capable to convert these mycotoxins to DON through hydrolysis of the acetyl moiety [69]. A biodegradation of modified mycotoxins by microorganisms is also assumed by Jensen et al. [70] during the ensiling process of forage maize due to the fact that anaerobic microorganisms (especially LAB) are responsible for the fermentation process [52,71,72]. Based on the outcome of the study of Jensen et al. [70], ensiling of forage maize result in a significant additional parent DON load solely arising from the degradation of DON3G (64% degradation into parent DON) and 3+15-AcDON (69% degradation into parent DON). Their results demonstrate that the sum of DON equivalents (DON + DON3G + 3+15-AcDON; µmol/kg) remains stable during the whole ensiling process and, therefore, a significant additional DON exposure must be expected after ensiling. In consequence, the determination of DON alone without consideration of DON3G and 3- and 15-AcDON is inadequate for assessing the possible total mycotoxin contamination in forage maize. The guidance value for DON in complementary and complete feeding stuffs of 5000 µg/kg might be exceeded even if the analysed concentrations of DON alone were below that value assuming a conversion of DON3G and 3+15-AcDON into parent DON (e.g., by ensiling). Our results demonstrate that the guidance value for DON can be exceeded more often when additional DON arising from DON3G and 3+15-AcDON is considered, increasing from 9% (measured DON alone) to 18%. Therefore, modified forms of DON ought to be considered by legislation together with their parent form to avoid animal health risks.

While the frequent occurrence of ZEN in forage maize samples is well documented [10,13,14,56,57], information about the occurrence of its modified form α-ZEL is rare. Although concentrations of α-ZEL reached relatively small proportions in relation to their measured parent ZEN concentrations (6.2 mol% averaged over all forage maize samples), considerable amounts of α-ZEL were determined in some samples also reaching higher α-ZEL/ZEL ratios. Therefore, our findings are of particular concern for animal health due to the fact that α-ZEL exerts a much higher oestrogenic activity than ZEN [24,73]. According to Frizzell et al. [73], α-ZEL was 70 times more potent than its parent form ZEN. In contrast to DON3G, 3- and 15-AcDON, ZEN, and its metabolite α-ZEL remain stable during ensiling of forage maize [70,74]. Hence, the presence of the highly oestrogenic ZEN metabolite α-ZEL increases the overall toxicity of ZEN-contaminated maize silage. To avoid an underestimation of α-ZEL in forage maize and, thus, the risk of hyper-oestrogenic effects in livestock, α-ZEL needs to be considered in routine mycotoxin analyses together with its parent form ZEN. In addition to DON3G and 3- and 15-AcDON, the occurrence of α-ZEL in products intended for animal feeding should also be regulated by legislation.

## 4. Conclusions

The consistent co-occurrence of DON and ZEN and its modified forms DON3G, 3- and 15-AcDON, and α-ZEL in considerable concentrations, the positive correlations between mycotoxin concentrations and the partly high relative proportions of modified mycotoxins in relation to parent DON and ZEN (especially DON3G/DON and 3+15-AcDON/DON) shown in our study, highlight the importance of modified mycotoxins in unprocessed forage maize. In view of practical feed control, DON3G, 3- and 15-AcDON, and α-ZEL can contribute substantially to the overall toxicity (e.g., directly or indirectly by conversion into parent form). We, therefore, conclude that these modified mycotoxins should be analysed in future mycotoxin monitoring programs together with DON and ZEN in forage maize to avoid animal health risks. Furthermore, the guidance value for DON and ZEN in products intended for animal feeding set in Commission Recommendation 2006/576/EC may be too high and should be reconsidered, e.g., by including a sum of mycotoxins consisting of parent mycotoxin and its co-occurring modified forms to avoid an underestimation of the actual mycotoxin contamination in forage maize.

## 5. Materials and Methods

### 5.1. Forage Maize Sampling

Forage maize samples were collected at harvest from eight locations in Schleswig-Holstein (Northern Germany) in 2017 (Table 4). This federal state between the Baltic and North Sea is characterized by maritime weather conditions, with an average annual temperature of 8.9 °C and an annual precipitation of 823 L/m^2^ [75]. In 2017, arable crops were grown on 651,000 ha with winter wheat and forage maize as dominant crops in crop rotation, accounting for 28.9% and 24.7% of the arable land, respectively, followed by winter oilseed rape (14.9%) and winter barley (10.3%) [76].

Within our survey, forage maize was cultivated in a crop rotation consisting of two to three crops at four of the sampled locations, whereas forage maize was the only crop in crop rotation (continuous maize) at the remaining locations (Table 4). Maize preceded forage maize at five of the eight locations. Soil cultivation by ploughing was the most commonly used cultivation practice followed by reduced cultivation tillage. In summary, these locations can be characterized as representative for the cultivation of forage maize based on the illustrated agronomic practices and large acreages of forage maize within the region surveyed.

Four cultivars of forage maize, namely “SY Werena” (=Cv I), “SY Kardona” (Cv II), “Colisee” (Cv III), and “Niklas” (Cv IV), were cultivated at all locations in a randomized complete block design with four replications (=field plots) per cultivar (at L2 and L7 three replications per cultivar). Forage maize was sown at the end of April with a sowing density of 10 plants/m^2^. Each field plot had a size of 30 m^2^ (3 × 10 m) and four rows per plot (row spacing 0.75 m). At all locations, trials were integrated in farmers’ fields without fungicide treatment or artificial *Fusarium* inoculation. Fertilization as well as weed management was conducted according to agricultural practice. Forage maize was harvested with a forage harvester (inner two rows) at mid-October with a dry matter content between 30% and 35%. A sample of 1000 g were taken from each plot and a total of 120 forage maize samples were analysed for their mycotoxin contamination. Sowing, crop management and harvesting were carried out in cooperation with the Chamber of Agriculture of Schleswig-Holstein.

### 5.2. Analysis of Mycotoxins

Sample preparation, mycotoxin extraction, and quantitative determination using liquid chromatography-high resolution mass spectrometry (LC-HRMS) were performed according to a validated method for the simultaneous quantitative determination of *Fusarium* mycotoxins in forage maize and maize silage described by Jensen et al. [77]. Forage maize samples were analysed for their concentrations of DON, DON3G, 3- and 15-AcDON, ZEN, α- and β-ZEL. Using the applied method, the isomers 3- and 15-AcDON could not be chromatographically separated, wherefore the sum of both (3+15-AcDON) were declared in this study. Detection limits (CCα) and detection capabilities (CCβ) of the analysed *Fusarium* mycotoxins in forage maize were 75 and 141 µg/kg for DON, 17 and 29 µg/kg for DON3G, 18 and 28 µg/kg for 3+15-AcDON, 36 and 60 µg/kg for ZEN, 16 and 26 µg/kg for α-ZEL, 73 and 108 µg/kg for β-ZEL, respectively.

### 5.3. Determination of Ratios between DON3G and DON, 3+15-AcDON and DON, α-ZEL and ZEN

For the determination of mycotoxin ratios between DON3G and DON, 3+15-AcDON and DON, α-ZEL and ZEN, data were converted from mass to molar concentrations through division by molar mass (DON: 296.31 g/mol; DON3G: 458.46 g/mol; 3- and 15-AcDON: 338.35 g/mol; ZEN: 318.36 g/mol; α-ZEL: 320.39 g/mol). Ratios were calculated as DON3G mol/DON mol × 100 (=DON3G/DON), 3+15-AcDON mol/DON mol × 100 (=3+15-AcDON/DON), α-ZEL mol/ZEN mol × 100 (=α-ZEL/ZEN) and were given in mol%. Finally, the ratios DON3G/DON and 3+15-AcDON/DON were summarized as the ratio 3+15-AcDON + DON3G/DON.

### 5.4. Statistical Analysis

Statistical analysis was done for concentrations of the several mycotoxins (DON, DON3G, 3+15-AcDON, ZEN, α-ZEL) and ratios (DON3G/DON, 3+15-AcDON/DON, 3+15-AcDON + DON3G/DON, α-ZEL/ZEN) by use of the statistical software R, version 4.0.1 (R Foundation for Statistical Computing, Vienna, Austria) [78]. For both, a mixed model was used [79,80,81,82] with location (L1–L8) as random factor. The model for the concentrations included the cultivar (“Cv I”, “Cv II”, “Cv III”, and “Cv IV”) and mycotoxin (DON, ZEN, DON3G, 3+15-AcDON, α-ZEL), as well as their interaction term as fixed factors. Additionally, the correlations of the measurement values from the same plot (due to the several types of mycotoxin) were also taken into account. We used a “log+1”-transformation for the measurement values because of their skewness. The model for the ratios included the cultivar as fixed factor. For both, concentrations and ratios, the residuals were assumed to be approximately normally distributed and to be heteroscedastic. These assumptions are based on a graphical residual analysis. Based on those models, a Pseudo *R^2^* was calculated [83] and an analysis of variances (ANOVA) was conducted, followed by multiple contrast tests [84]. For the concentrations, the several mycotoxins within the same cultivar, and cultivars within the same mycotoxin were compared. For the ratios, the cultivar means were tested versus zero and versus each other. Statistical significance was evaluated at *p* ≤ 0.05.

## Figures and Tables

**Table 1:** Incidences (%) and concentrations (mean, mean positive, median, minimum, maximum; µg/kg) of DON, DON3G, 3+15-AcDON, ZEN, α- and β-ZEL in forage maize samples summarized for the four investigated cultivars. *n* = 120.

Mycotoxin	Incidence (%) ^a^	Mean (µg/kg) ^b^	Mean Positive (µg/kg) ^c^	Median (µg/kg) ^b^	Min–Max (µg/kg)	Samples ≥Guidance Value (%) ^d^
DON	100	2165	2165	1391	<CCβ–10,972	9 (11/120)
DON3G	100	537	537	348	95–3038	− ^f^
3+15-AcDON	97	516	534	346	<CCα–2237	− ^f^
ZEN	96	819	862	426	<CCα–3910	46 (55/120)
α-ZEL	59	44	74	26	<CCα–423	− ^f^
β-ZEL	32	n.a ^e^	n.a ^e^	n.a ^e^	<CCα–203	− ^f^

^a^ Percentage of positive samples (≥CCα/total samples). ^b^ For calculation, concentrations below CCα were set to zero, whereas concentrations between CCα and CCβ were set to CCβ. ^c^ Only positive samples were considered. For calculation, concentrations <CCβ were set to CCβ. ^d^ Guidance values for DON (5000 µg/kg) and ZEN (500 µg/kg) in complementary and complete feeding stuffs [27]. n.a ^e^ = not applicable. – ^f^ = Guidance value not available. CCα = detection limit. CCβ = detection capability. DON = deoxynivalenol; DON3G = deoxynivalenol-3-glucoside; 3+15-AcDON = sum of 3- and 15-acetyl-deoxynivalenol; ZEN = zearalenone; α-ZEL = α-zearalenol; β-ZEL = β-zearalenol.

**Table 2:** Incidences (%) and concentrations (mean, mean positive, median, minimum, maximum; µg/kg) of DON, DON3G, 3+15-AcDON, ZEN, α- and β-ZEL in forage maize samples of four different cultivars (Cv I–IV). Different letters describe significant differences (*p* ≤ 0.05). (i) between cultivars within the same mycotoxin (capital letters) and (ii) between the several mycotoxins within the same cultivar (small letters). *n* = 120.

Cv ^a^	Mycotoxin	Incidence (%) ^b^	Mean (µg/kg) ^c^	Mean Positive (µg/kg) ^d^	Median (µg/kg) ^c^	Min–Max (µg/kg)	Samples ≥Guidance Value (%) ^e^
“Cv I” (*n* = 30)	DON	100	2486 Aa	2486	1651	466–10,972	13 (4/30)
	DON3G	100	507 ABb	507	300	119–1911	− ^g^
	3+15-AcDON	100	472 ABb	472	281	29–1837	− ^g^
	ZEN	100	785 Ab	785	621	<CCβ–2334	53 (16/30)
	α-ZEL	70	64 Ac	92	26	<CCα–423	− ^g^
	β-ZEL	53	n.a ^f^	n.a ^f^	n.a ^f^	<CCα–163	− ^g^
“Cv II” (*n* = 30)	DON	100	2673 Aa	2673	1778	467–9305	13 (4/30)
	DON3G	100	776 Ab	776	511	112–3038	− ^g^
	3+15-AcDON	100	655 Ab	655	524	112–2049	− ^g^
	ZEN	100	1055 Ab	1055	558	124–3076	53 (16/30)
	α-ZEL	57	44 Ac	78	26	<CCα–188	− ^g^
	β-ZEL	30	n.a ^f^	n.a ^f^	n.a ^f^	<CCα–118	− ^g^
“Cv III” (*n* = 30)	DON	100	2054 ABa	2054	1414	<CCβ–9638	7 (2/30)
	DON3G	100	526 ABb	526	328	95–1286	− ^g^
	3+15-AcDON	93	559 ABb	599	378	<CCα–2087	− ^g^
	ZEN	93	666 Ab	714	397	<CCα–2881	43 (13/30)
	α-ZEL	57	31 Ac	55	26	<CCα–199	− ^g^
	β-ZEL	23	n.a ^f^	n.a ^f^	n.a ^f^	<CCα–119	− ^g^
“Cv IV” (*n* = 30)	DON	100	1449 Ba	1449	991	<CCβ–7104	3 (1/30)
	DON3G	100	339 Bb	339	253	100–795	− ^g^
	3+15-AcDON	93	378 Bb	405	267	<CCα–2237	− ^g^
	ZEN	90	768 Ab	886	225	<CCα–3910	33 (10/30)
	α-ZEL	53	36 Ac	68	26	<CCα–324	− ^g^
	β-ZEL	20	n.a ^f^	n.a ^f^	n.a ^f^	<CCα–203	− ^g^

^a^ Cv = Cultivar. ^b^ Percentage of positive samples (≥CCα/total samples). ^c^ For calculation, concentrations below CCα were set to zero, whereas concentrations between CCα and CCβ were set to CCβ. ^d^ Only positive samples were considered. For calculation, concentrations <CCβ were set to CCβ. ^e^ Guidance values for DON (5000 µg/kg) and ZEN (500 µg/kg) in complementary and complete feeding stuffs [27]. n.a ^f^ = not applicable. – ^g^ = Guidance value not available. CCα = detection limit. CCβ = detection capability. DON = deoxynivalenol; DON3G = deoxynivalenol-3-glucoside; 3+15-AcDON = sum of 3- and 15-acetyl-deoxynivalenol; ZEN = zearalenone; α-ZEL = α-zearalenol; β-ZEL = β-zearalenol. Note: The significance conclusions are based on log-transformed mycotoxin concentrations due to the skewed distribution of the measured values.

**Table 3:** Pairwise Spearman rank correlation coefficients (*r*) between mycotoxin concentrations of DON, DON3G, 3+15-AcDON, ZEN, and α-ZEL (µg/kg) in forage maize samples of four different cultivars (Cv I–IV). *n* = 120.

Correlation	Cultivar				
	“Cv I”	“Cv II”	“Cv III”	“Cv IV”	All Cultivars
DON vs. DON3G	0.907	0.877	0.926	0.833	0.866
DON vs. 3+15-AcDON	0.892	0.888	0.929	0.952	0.915
DON3G vs. 3+15-AcDON	0.883	0.799	0.866	0.815	0.841
DON vs. ZEN	0.704	0.667	0.788	0.696	0.714
DON3G vs. ZEN	0.686	0.579	0.751	0.633	0.662
3+15-AcDON vs. ZEN	0.712	0.631	0.689	0.640	0.668
DON vs. α-ZEL	0.610	0.600	0.615	0.443	0.567
DON3G vs. α-ZEL	0.627	0.537	0.517	0.334	0.504
3+15-AcDON vs. α-ZEL	0.661	0.582	0.647	0.471	0.590
ZEN vs. α-ZEL	0.663	0.593	0.635	0.584	0.619

DON = deoxynivalenol; DON3G = deoxynivalenol-3-glucoside; 3+15-AcDON = sum of 3- and 15-acetyl-deoxynivalenol; ZEN = zearalenone; α-ZEL = α-zearalenol.

**Table 4:** Coordinates and agronomic practices (crop rotation, previous crop, soil cultivation) of the eight trial locations in Northern Germany in 2017. Coordinate System: WGS 1984 Web Mercator Auxiliary Sphere (EPSG 3857).

Location (Abbreviation)	Coordinates (EPSG 3857)		Crop Rotation ^a^	Previous Crop ^a^	Soil Cultivation
	*x*	*y*			
Barkhorn (L1)	1,073,958	7,211,748	Continuous FM	FM	Reduced tillage
Futterkamp (L2)	1,183,896	7,225,601	OR-WW-FM-FM	FM	Plough
Leezen (L3)	1,139,549	7,146,520	WR-FM	WR	Plough
Medelby 1 ^b^ (L4)	1,022,547	7,325,312	Continuous FM	FM	Plough
Medelby 2 ^b^ (L5)	1,022,547	7,325,312	Continuous FM	FM	Reduced tillage
Scholderup (L6)	1,076,900	7,278,022	Continuous FM	FM	Plough
Schuby (L7)	1,051,158	7,269,157	Potato-WR-FM	WR	Plough
Wallsbüll (L8)	1,030,547	7,318,384	WW-FM-WR	WW	Plough

^a^ FM = Forage maize, OR = Winter oilseed rape, WR = Winter rye, WW = Winter wheat. ^b^ Two fields at this location which only differ in the type of soil cultivation.

## Data Availability

Data available upon request.

## Supplementary Materials

The following are available online at https://www.mdpi.com/2072-6651/13/2/110/s1, Figure S1: Relationship between measured DON concentrations (µg/kg) and ratios of (a) DON3G/DON (mol%), and (b) 3+15-AcDON/DON (mol%) in forage maize samples of four different cultivars (Cv). DON = deoxynivalenol; DON3G = deoxynivalenol-3-glucoside; 3+15-AcDON = sum of 3- and 15-acetyl-deoxynivalenol. *n* = 120, Figure S2: Relationship between measured ZEN concentrations (µg/kg) and ratios of α-ZEL/ZEN (mol%) in forage maize samples of four different cultivars (Cv) (a –d). ZEN = zearalenone; α-ZEL = α-zearalenol. *n* = 120, Figure S3: Concentrations (µg/kg) of ZEN + α-ZEL in ZEN positive forage maize samples of four different cultivars (Cv) (a–d). Samples were sorted by increasing concentration of ZEN + α-ZEL for each cultivar separately. The guidance value for ZEN in complementary and complete feeding stuffs (500 µg/kg) is shown as yellow line. ZEN = zearalenone; α-ZEL = α-zearalenol. *n* = 120.
