# Supplementary material for: Occurrence of Fusarium Mycotoxins and Their Modified Forms in Forage Maize Cultivars
Source: Toxins (Basel). 2021 Feb 2;13(2):110. doi: 10.3390/toxins13020110 (PMC7913079; doi:10.3390/toxins13020110)
Supplement: Supplementary file 1 [file toxins-13-00110-s001.pdf]

Supplementary Materials: Occurrence of *Fusarium* Mycotoxins and Their Modified Forms in Forage Maize Cultivars

Tim Birr, Tolke Jensen, Nils Preußke, Frank D. Sönnichsen, Marthe De Boevre, Sarah De Saeger, Mario Hasler, Joseph-Alexander Verreet and Holger Klink

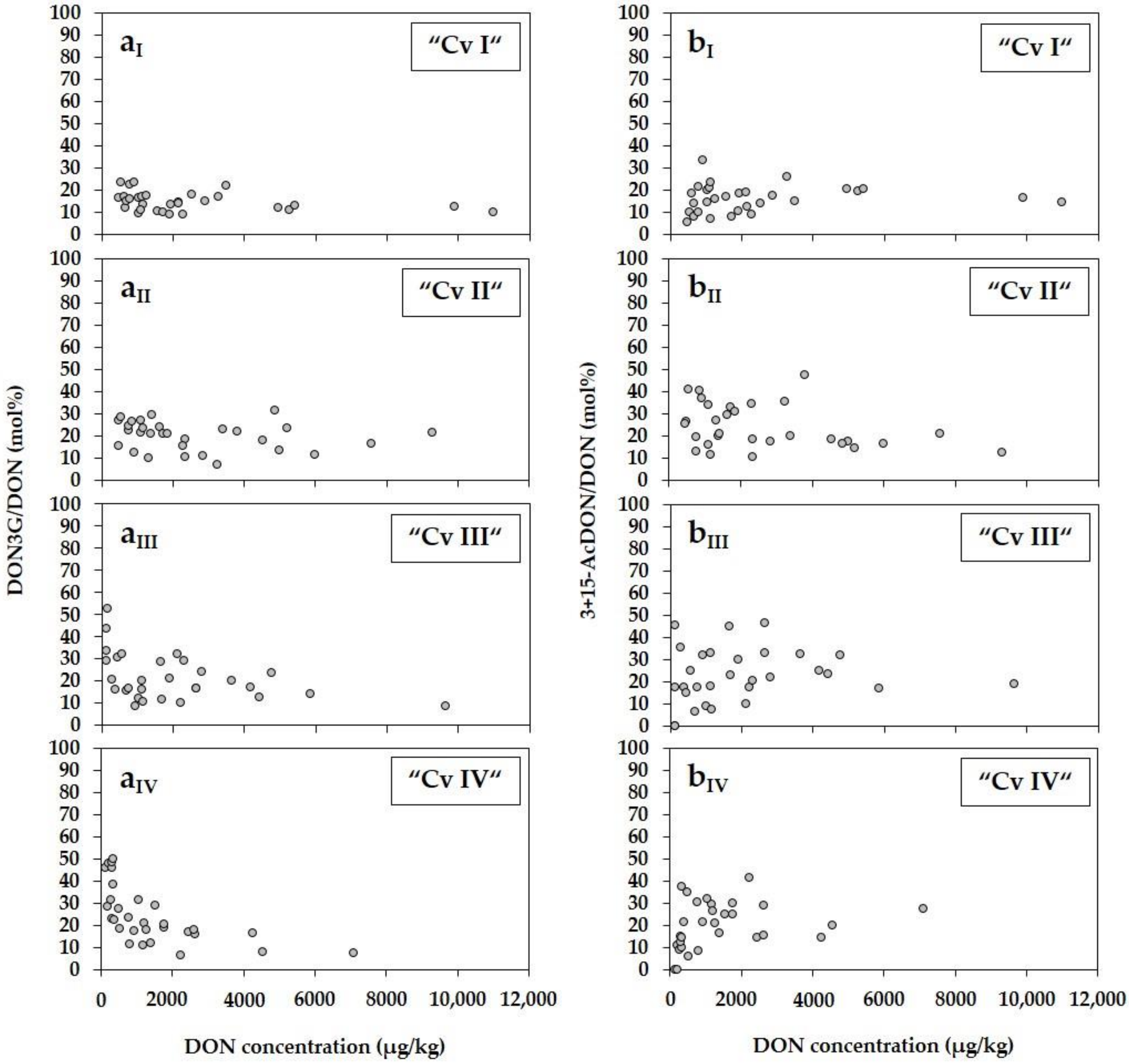

Figure S1. Relationship between measured DON concentrations (µg/kg) and ratios of (**a**) DON3G/DON (mol%), and (**b**) 3+15-AcDON/DON (mol%) in forage maize samples of four different cultivars (Cv). DON = deoxynivalenol; DON3G = deoxynivalenol-3-glucoside; 3+15-AcDON = sum of 3- and 15-acetyl-deoxynivalenol. *n* = 120.

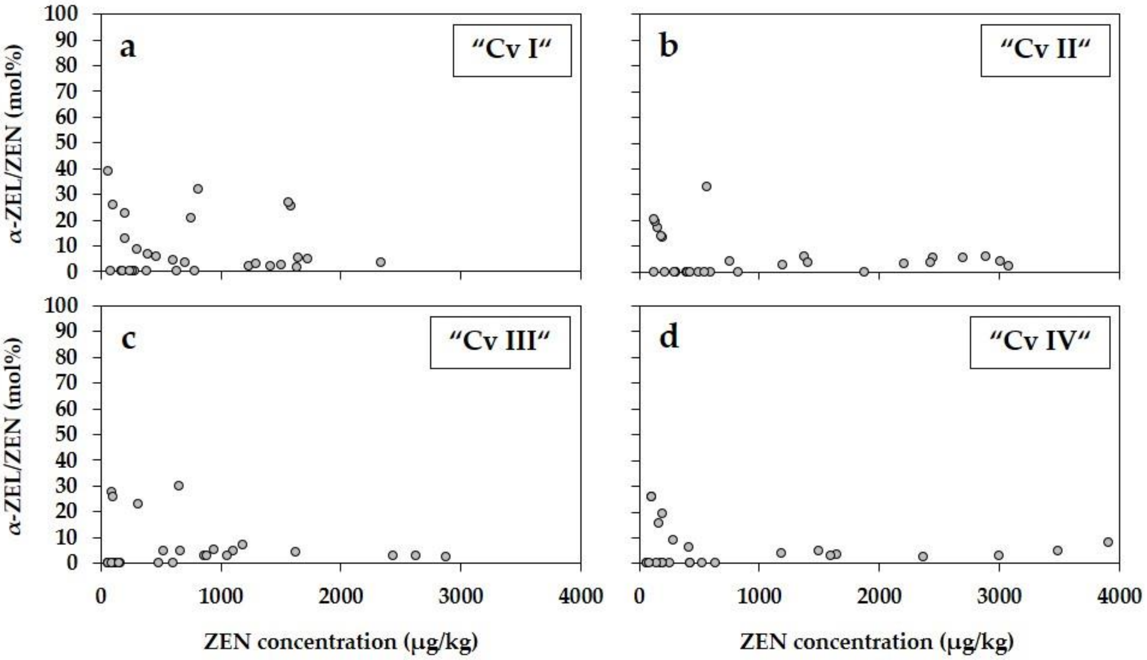

Figure S2. Relationship between measured ZEN concentrations (µg/kg) and ratios of α-ZEL/ZEN (mol%) in forage maize samples of four different cultivars (Cv) (**a**–**d**). ZEN = zearalenone; α-ZEL = α-zearalenol. n = 120.

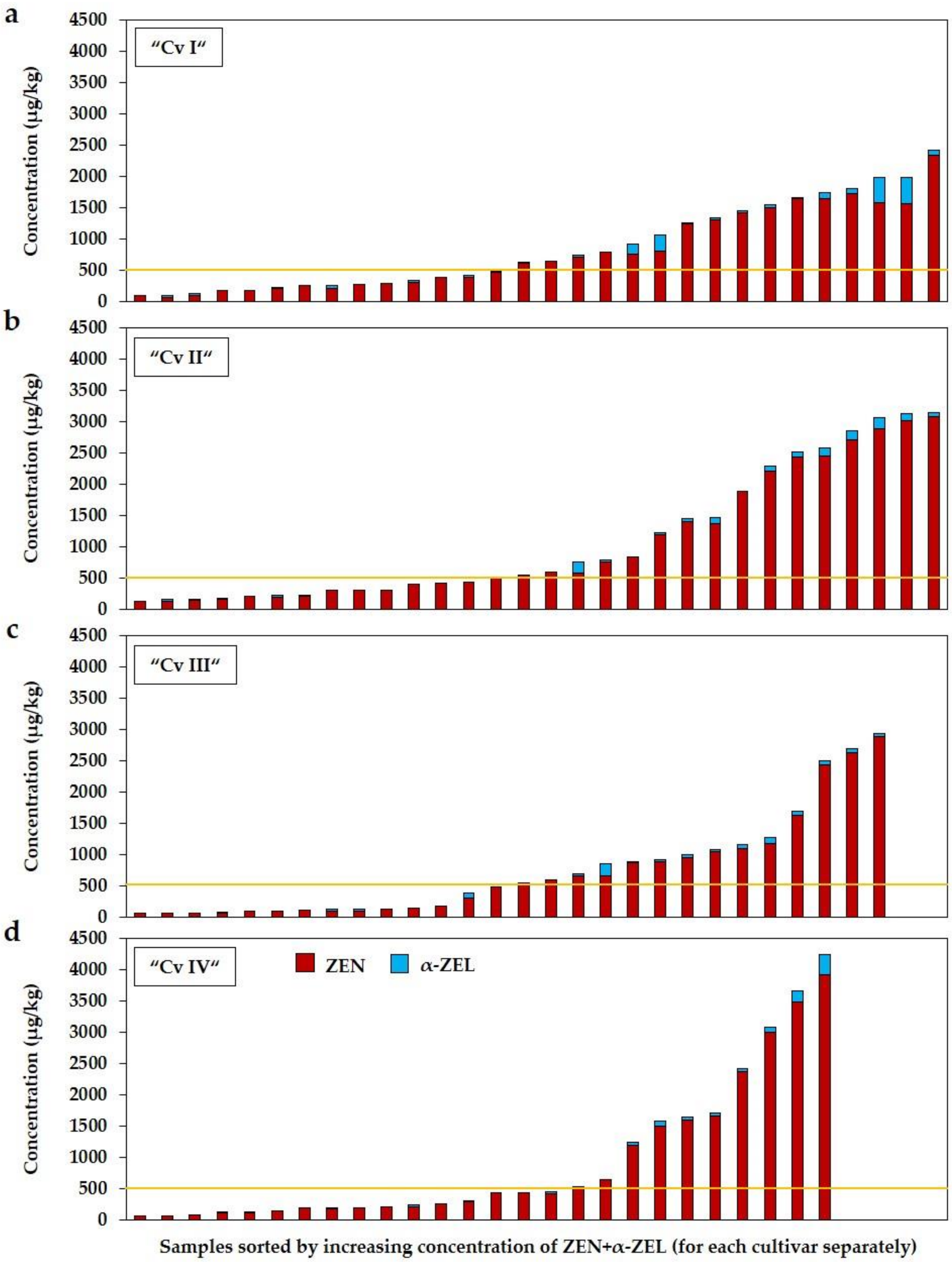

Figure S3. Concentrations (µg/kg) of ZEN+α-ZEL in ZEN positive forage maize samples of four different cultivars (Cv) (**a**–**d**). Samples were sorted by increasing concentration of ZEN+α-ZEL for each cultivar separately. The guidance value for ZEN in complementary and complete feeding stuffs (500 µg/kg) is shown as yellow line. ZEN = zearalenone; α-ZEL = α-zearalenol. *n* = 120.
